# A DDoS Detection Method Based on Feature Engineering and Machine Learning in Software-Defined Networks

**DOI:** 10.3390/s23136176

**Published:** 2023-07-05

**Authors:** Zhenpeng Liu, Yihang Wang, Fan Feng, Yifan Liu, Zelin Li, Yawei Shan

**Affiliations:** 1 School of Electronic Information Engineering, Hebei University, Baoding 071002, China; lzp@hbu.edu.cn (Z.L.); 20217018082@stumail.hbu.edu.cn (Y.W.);; 2 Information Technology Center, Hebei University, Baoding 071002, China; 3 School of Cyberspace Security and Computer, Hebei University, Baoding 071002, China

**Keywords:** software-defined networking, DDoS attacks, feature engineering, machine learning, binary grey wolf optimization algorithm

## Abstract

Distributed denial-of-service (DDoS) attacks pose a significant cybersecurity threat to software-defined networks (SDNs). This paper proposes a feature-engineering- and machine-learning-based approach to detect DDoS attacks in SDNs. First, the CSE-CIC-IDS2018 dataset was cleaned and normalized, and the optimal feature subset was found using an improved binary grey wolf optimization algorithm. Next, the optimal feature subset was trained and tested in Random Forest (RF), Support Vector Machine (SVM), K-Nearest Neighbor (k-NN), Decision Tree, and XGBoost machine learning algorithms, from which the best classifier was selected for DDoS attack detection and deployed in the SDN controller. The results show that RF performs best when compared across several performance metrics (e.g., accuracy, precision, recall, F1 and AUC values). We also explore the comparison between different models and algorithms. The results show that our proposed method performed the best and can effectively detect and identify DDoS attacks in SDNs, providing a new idea and solution for the security of SDNs.

## 1. Introduction

With the development of emerging technologies such as cloud computing and big data, network traffic and dependency on networks have increased. Especially in 2020, the global COVID-19 pandemic increased online work, learning, and entertainment, placing higher demands on network stability and security. The problems with traditional network models have become more significant and cannot effectively meet users’ needs. Software-defined networking (SDN) has become the preferred network technology due to its flexibility, programmability, dynamism, and simplicity [1,2,3].

**Figure 1:**
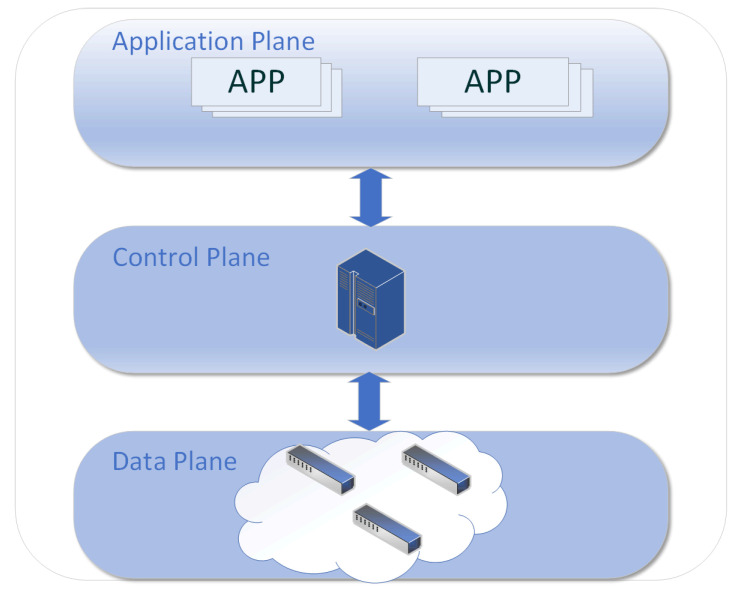
SDN architecture.

SDN uses a design that separates the control and data planes, as shown in Figure 1. There are mainly three planes: the application plane, the control, and the data plane. Devices such as switches and routers are placed in the data plane, which is programmed and managed by the control plane. Interaction with the controller takes place via a southbound interface. The control plane is responsible for managing the transport devices on the data plane, and the controller, which is the brain of the network, is located on this plane. The application plane consists of many SDN applications that are of interest to the user at the time of the SDN application. It can interact with the SDN controller through a northbound interface, i.e., these applications can submit the network behavior that needs to be requested to the controller in a programmable way. The controller globally controls the network topology, decentralizing complex traffic processing from switches and routers to the data plane. This increases the network’s scalability, controllability, and programmability while significantly enhancing network traffic management and resource-utilization efficiency. Nevertheless, network security remains a critical issue in SDN applications [4]. Distributed denial-of-service (DDoS) attacks pose significant security threats to networks. Attackers utilize many spurious requests to exhaust the target host’s resources, rendering it unable to provide regular service. These highly targeted and stealthy attacks have low launch costs, making them increasingly serious threats.

Furthermore, attackers employ increasingly sophisticated methods, making DDoS attack detection more challenging to counter. If the attack targets the controller, it cannot respond to requests, causing network failure. If the attack targets the data plane, it will cause vast amounts of meaningless traffic resulting from the DDoS attack consuming too much network bandwidth and processing resources. Such traffic affects the normal forwarding of regular traffic while causing the SDN controller to issue unnecessary flow tables to switches for routing, which will utilize SDN switch storage space, further increasing the data plane’s burden. Hence, effectively detecting and mitigating DDoS attacks has become a research focus in the context of SDN.

The primary detection methods are commonly categorized as statistics-based and machine-learning-based [5,6]. Flow modeling is employed in statistics-based detection methods to analyze specific network protocols or application layer information to detect DDoS attacks. These methods establish expected behavior or traffic models and detect anomalies from attack traffic. Although these methods are simple and efficient, they are unable to handle new attack scenarios and require manual setup for each new attack type [7]. In contrast, machine-learning-based DDoS detection is a more intelligent technique, using machine learning algorithms to learn normal traffic patterns, detect anomalous traffic patterns, and identify DDoS attacks automatically. This method can adapt to new attack scenarios and uncover hidden attack patterns from complex network environments. However, it requires vast datasets and computing resources for training and testing algorithms. Combining the advantage of SDN technology, the machine-learning-based approach can achieve DDoS detection and effective mitigation through automation, efficient response, and Deep Learning [8] in the network security field.

Feature engineering is essential in building a machine learning model because it retains the most valuable information while eliminating redundant and irrelevant information [9,10,11]. Consequently, handling the dataset effectively is a critical challenge facing researchers. The quality of data and features determines machine learning performance, while models and algorithms only approach this ceiling. Therefore, researchers must strive for a high-quality dataset to improve the accuracy of intrusion detection [12,13]. This paper proposes a feature-engineering- and machine-learning-based intrusion detection system. We begin by employing an improved binary grey wolf optimization algorithm to select the optimal features from the dataset. Subsequently, we compare five machine learning classification algorithms using the optimal feature subset and deploy the optimal classification algorithm in the SDN’s controller for DDoS attack detection. The main contributions of this paper are summarized as follows:

1. Perform a series of feature engineering on the dataset, including data cleaning, transformation, and feature extraction and selection using an improved binary grey wolf optimization algorithm to obtain the optimal feature subset.
2. Train and test five different machine learning classifiers with the optimal feature subset and compare the results of this work with the classification results using the original dataset.
3. Deploy the optimal classifier in the SDN controller for DDoS detection and take appropriate action if an attack is detected.
4. Evaluate, validate, and compare the proposed approach with existing research.

The organization of this paper is as follows: Section 1 provides an introduction. Section 2 discusses previous research on DDoS detection in SDN. Section 3 presents relevant background knowledge. Section 4 proposes the method used in this paper. Section 5 analyzes and discusses the experimental results. Finally, Section 6 concludes the paper.

## 2. Related Work

This section reviews the latest research progress on DDoS detection in SDN using feature-engineering-based methods.

Many researchers have used existing feature selection algorithms in the field of feature selection. Polat et al. [14] collected real-time traffic data from SDNs under normal conditions and DDoS attacks, respectively, as datasets. Then, they extracted features using filter-based, wrapper-based, and embedded feature selection methods. SVM, Naive Bayes (NB), Artificial Neural Network (ANN), and k-NN classification models were trained and tested using the dataset after feature extraction. The test results revealed that the k-NN classifier achieved the highest accuracy, 98.3%, among all classifiers for detecting DDoS attacks. Beitollahi et al. [15] employed a radial basis function (RBF) neural network and cuckoo search (CS) algorithm to detect DDoS attacks. They first utilized a genetic algorithm (GA) to search for the optimal feature subset for data collection. Then, the RBF neural network was trained with the optimal feature subset and the CS optimization algorithm. A comparison between this method and the k-NN, Bootstrap aggregation, SVM, MLP, and recurrent neural network (RNN) methods was conducted. Experimental results revealed that this method outperformed previous methods in detecting DDoS traffic. Mishra et al. [16] categorized DDoS attacks and trained and predicted them based on various criteria. They pre-processed the CICDoS2019 dataset by cleaning and transforming it and optimized the features using the Extra Tree Classifier, which produced 25 optimal features. They applied six different machine learning algorithms and found that the AdaBoost Classifier achieved the highest accuracy of 99.87%. Aamir et al. [17] presented a strategic framework that combines feature engineering and machine learning. They applied the t-statistics test, Chi2, and information gain for selecting significant features of various dimensions from the dataset. Then, they employed five distinct supervised machine learning algorithms to compare with the three datasets. The results indicated that the k-NN algorithm achieved the highest overall performance.

Maheshwari et al. [18] developed a testing platform employing Mininet, POX controller, and multiple datasets. They used a novel hybrid meta-heuristic optimization algorithm (BHO) to identify the optimal feature set. They validated an ensemble method with six basic classifiers, including two SVMs, two Random Forests, and two Gradient Boosting Machines. Akgun et al. [19] utilized the CIC-IDDoS2019 dataset to select 40 essential features using the information gain attribute evaluation algorithm. The researchers employed a Convolutional Neural Network (CNN) model with a one-dimensional convolutional layer for detecting DDoS attacks. Karatas et al. [20] employed SMOTE (Synthetic Minority Oversampling Technique), a data synthesis model, to alleviate dataset imbalance. The minority class was augmented to reach an average dataset size using SMOTE. Classification tasks were performed using k-NN, RF, Gradient Boosting, AdaBoost, Decision Tree, and Linear Discriminant Analysis algorithms. The experiment’s findings indicate that this methodology enhances the detection rate of rare intrusions.

Using neural networks for feature selection is a viable option. Polat et al. [21] presented a new network architecture that incorporates Long Short-Term Memory (LSTM) and Gated Recurrent Unit (GRU) layers following the input layer, each with a corresponding dropout layer. These parallel layers are combined into an additional layer that extracts chosen features, then classified using SVM on the final feature vector obtained from the additional layer. Thangasamy et al. [22] proposed a DDoS attack detection method that utilizes a hybrid LSTM model and deep belief network-based feature extraction. The deep belief network method extracts features from the NSL-KDD dataset, which are used to identify DDoS attacks via LSTM neural networks optimized via a particle swarm optimization (PSO) algorithm. This approach is highly effective in accurately predicting regular network traffic while detecting anomalies caused by DDoS attacks. DORA et al. [10] employed a CNN and an optimized LSTM ensemble for DDoS detection. They first utilized the grey wolf optimization algorithm (CP-GWO) for feature selection. After selecting optimal features, they utilized CNN for feature learning and extracted the second pooling layer’s features for detection. Finally, they used the optimized LSTM for detection and maximizing detection accuracy by optimizing the hidden neurons in the network.

Researchers often generate feature combinations based on the characteristics of DDoS attack traffic. Zhou et al. [23] analyzed the characteristics of various DDoS attacks and proposed five new features from packets with different characteristics. These features can detect multiple types of DDoS attacks, including mixed attacks. Chouhan et al. [24] extracted seven features from SDNs’ normal and DDoS attack traffic. They constructed a dataset using these seven-tuple feature vectors to train and test five classifiers. The performance evaluation was based on accuracy, recall, precision, F1 score, FAR, and test time. Dong et al. [25] proposed four features, namely, flow length, flow duration, flow size, and flow ratio, along with two methods for detecting DDoS attacks in SDNs. One of the methods detects DDoS attacks through the degree of attack, while the other method uses an improved machine-learning-based k-NN algorithm. Ahuja et al. [26] created an SDN dataset using Mininet, which simulated real-time scenarios and extracted 23 features. Eight of the twenty-three features were used for classification, and the traffic was classified using SVC. The researchers used a Random Forest filter for classification afterwards. The accuracy of the method was as high as 98.8%.

Table 1 summarizes respective feature selection methods and classification models in related works.

## 3. Relevant Knowledge

This section details the basic algorithms used, such as the grey wolf optimization algorithms and binary grey wolf optimization algorithms.

### 3.1. Standard Grey Wolf Optimization Algorithm

**Figure 2:**
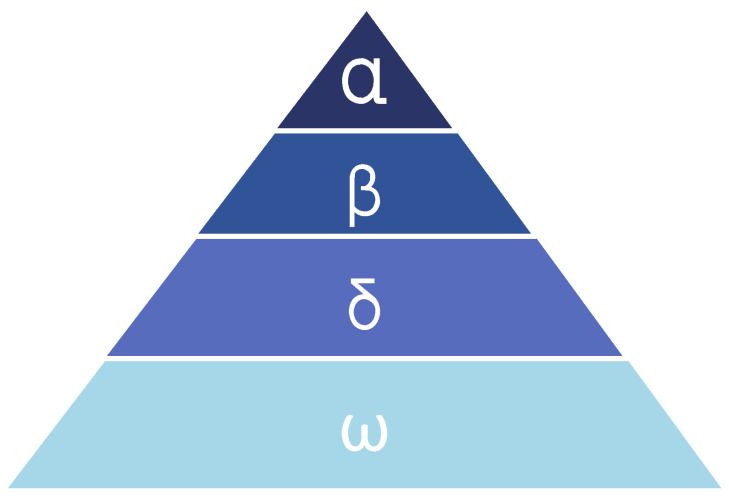
Hierarchy of grey wolves.

The grey wolf optimization algorithm (GWO) [27] is a typical swarm intelligence algorithm inspired by grey wolves’ hierarchical leadership and natural hunting mechanism, as shown in Figure 2. The method for seeking the optimal global solution is similar to that of other population-based intelligence optimization algorithms. However, the mathematical model of this algorithm is innovative and allows the grey wolf to locate another solution by searching around a solution in an n-dimensional search space, simulating grey wolves’ hunting and surrounding of prey. In addition, unlike other feature selection methods, it does not require threshold parameters to cut off irrelevant features.

To mathematically model the social hierarchy of grey wolves in GWO, four levels are defined: Alpha, Beta, Delta, and Omega. Each wolf represents a candidate solution, with Alpha, Beta, and Delta referring to the top three optimized solutions and Omega representing other wolves. The Omega updates their positions around Alpha, Beta, and Delta.

In GWO, wolves update their positions during hunting using the following formula to surround their prey:

(1) $$\vec{X}\left(t+1\right)=\vec{X_{p}}\left(t+1\right)-\vec{A}\cdot \vec{D}$$

(2) $$\vec{D}=\left|\vec{C}\cdot \vec{X_{p}}\left(t\right)-\vec{X}\left(t\right)\right|$$

In this formula, $\vec{X_{p}}$ represents the position vector of the prey, $\vec{X}$ represents the position vector of the grey wolf, t represents the current iteration, and $\vec{A}\;$ and $\;\vec{C}$ represent the coefficients. $\vec{A}$ and $\vec{C}$ are defined as follows:

(3) $$\vec{A}=2\vec{a}\cdot \vec{r_{1}}-\vec{a}$$

(4) $$\vec{C}=2\vec{r_{2}}$$

Vectors $r_{1}$ and $r_{2}$ are randomly generated from the interval $\left[0,\;1\right]$. a is a critical parameter that controls the utilization and exploration capabilities of the GWO algorithm, defined as a linearly decreasing parameter from 2 to 0 as iteration increases, defined as follows:

(5) $$a=2-2\cdot \frac{t}{T}$$

However, within the abstract search space, the exact location of the optimal prey cannot be determined. To mathematically model the hunting behavior of grey wolves, we assume that Alpha represents the best candidate solution, while Beta and Delta are the second and third solutions [28]. Update its position formula as defined below.

(6) $$\vec{X}\left(t+1\right)=\frac{\vec{X_{1}}+\vec{X_{2}}+\vec{X_{3}}}{3}$$

(7) $$\left\{\begin{array}{c} \vec{X_{1}}=\left|\vec{X_{\alpha }}-A_{1}\cdot \vec{D_{\alpha }}\right|\\ \vec{X_{2}}=\left|\vec{X_{\beta }}-A_{2}\cdot \vec{D_{\beta }}\right|\\ \vec{X_{3}}=\left|\vec{X_{\delta }}-A_{3}\cdot \vec{D_{\delta }}\right| \end{array}\right.$$

(8) $$\left\{\begin{array}{c} \vec{D_{\alpha }}=\left|\vec{C_{1}}\cdot \vec{X_{\alpha }}-\vec{X}\right|\\ \vec{D_{\beta }}=\left|\vec{C_{2}}\cdot \vec{X_{\beta }}-\vec{X}\right|\\ \vec{D_{\delta }}=\left|\vec{C_{3}}\cdot \vec{X_{\delta }}-\vec{X}\right| \end{array}\right.$$

$X_{1}$, $X_{2}$, and $X_{3}$ represent the positions that the Omega wolf needs to adjust based on the influence of Alpha wolf, Beta wolf, and Delta wolf, respectively. $\vec{D_{\alpha }}$, $\vec{D_{\beta }}$, and $\vec{D_{\delta }}$ represent the distances between Omega wolf and Alpha wolf, Beta wolf, and Delta wolf, respectively.

### 3.2. Binary Grey Wolf Optimization Algorithm

Grey wolves in the standard GWO algorithm optimize their position by continuously changing it to any location in space. However, feature selection is inherently a binary problem that requires solutions of a binary value of either 0 or 1. As a result, the standard GWO algorithm cannot solve this problem directly, necessitating the discretization of the GWO algorithm. The binary grey wolf optimization algorithm (BGWO) [29] represents each feature subset as a binary value, with each feature corresponding to a binary bit. Its goal is to find the optimal feature subset. In the optimization process of the algorithm, binary operations and evaluation functions are employed to assess the adaptability and quality of each feature subset.

Applying a transfer function, BGWO maps the optimization value of grey wolves from continuous space to discrete space. After updating, this optimizer forces the grey wolf position vector to convert into binary bits by utilizing the following equation:

(9) $$X_{i}^{d}\left(t\right)=\left\{\begin{array}{cc} 1 & if\;sigmoid\left(X_{i}^{d}\left(t\right)\right)>rand\\ 0 & otherwise \end{array}\right.$$

In this study, rand is a random number between 0 and 1 and $X_{i}^{d}\left(t\right)$ represents the position of the $wolf_{i}$ in the d dimension of the population during the t iteration. In this study, this is calculated using Equation (17). The calculation of the sigmoid is shown as follows:

(10) $$sigmoid\left(a\right)=\frac{1}{1+e^{-10\left(a-0.5\right)}}$$

The essence of the feature selection problem lies in seeking the minimum number of features while aiming for maximum classification accuracy. To take both aspects into account, the following equation is used as the fitness function:

(11) $$fitness=\alpha \frac{m}{M}+\beta \cdot error\;rate$$

Here, $\alpha +\beta =1\left(\beta >\alpha \right)$, where m and M represent the selected number of features and the total number of features, respectively; error rate describes the classification error rate of the k-NN classifier.

## 4. Methodology

**Figure 3:**
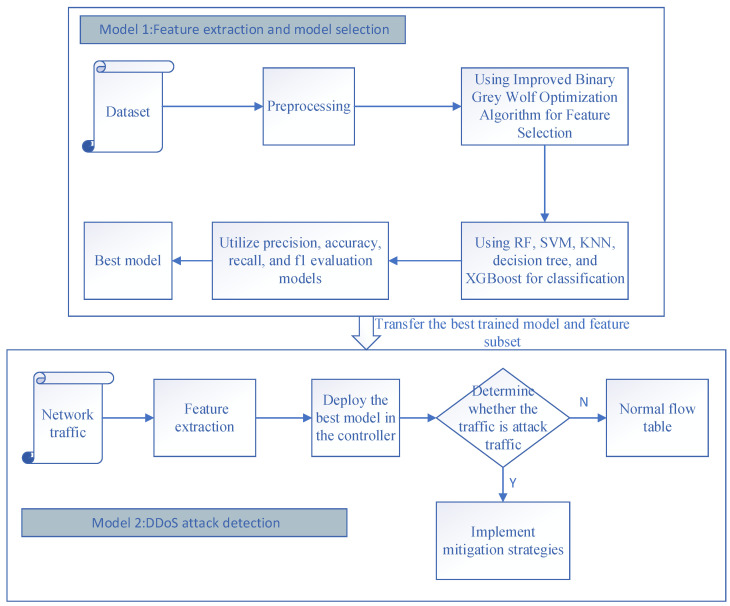
Flow of the proposed work.

This chapter focuses on a DDoS attack detection and defense scheme based on an improved binary grey wolf optimized feature extraction algorithm and machine learning in SDN. The flow of the proposed work in this paper is illustrated in Figure 3. It mainly consists of two major models: the feature extraction and model selection module and the DDoS attack detection module.

In module 1, the dataset is first preprocessed, and the optimal feature subset is obtained with feature selection using an improved binary grey wolf optimization algorithm; then, the selected feature subset is trained and tested using five supervised learning classifiers, namely, Support Vector Machine (SVM), Random Forest (RF), Decision Tree, XGBoost, and k-nearest Nearest Neighbor (k-NN) algorithms are used to train and test the selected feature subsets. Among them, the best-performing classifier is selected as the optimal classifier and finally deployed in the controller. Supervised learning was used because it offers several advantages, such as improving the clarity of the data and making training easier [30,31]. Subsequently, the optimal classification model and the best feature subset are passed to module 2. In module 2, network traffic is characterized and normalized according to the best feature subset. DDoS attack detection of SDN traffic is achieved by determining whether it is an attack traffic using the best classification model deployed in the controller. If any attack traffic is detected, the user is immediately notified, and the necessary mitigation strategies are implemented to ensure proper server functionality. If no malicious activity is detected, the flow table is usually dispatched.

### 4.1. Data Preprocessing and Feature Extraction

#### 4.1.1. Selection of the Dataset

**Figure 4:**
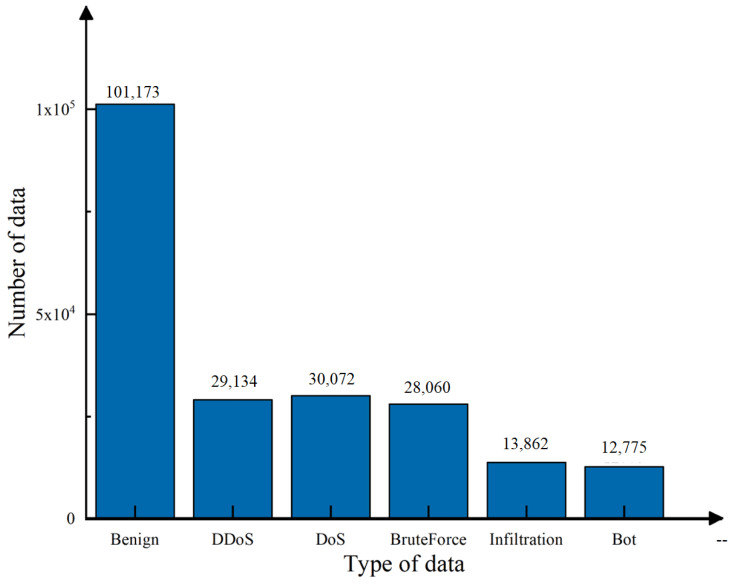
Distribution of data types in the dataset.

In this study, we utilized the CSE-CIC-IDS2018 dataset provided by the University of New Brunswick in Canada as our experimental dataset. It offers the following advantages: there are few duplicates, almost no uncertainty in the data, and the dataset is in CSV format to be used without processing. The dataset contains 79 features covering a wide range of attack types. Due to this dataset’s significant data volume imbalance, we selected an average of 210,000 sample data, including DDoS, DoS, Brute Force, Infiltration, and Bot attack types, as shown in Figure 4. Due to the variability in the characteristics of the different attack types, it may not be ideal to detect other attacks using only the selected characteristics of one attack type, so by including multiple types of attack data, we can ensure that we can detect the full range of attacks to ensure practical experimentation.

#### 4.1.2. Data Cleaning for the Dataset

While preparing a dataset, it is common to encounter many data quality issues such as Nan values, outliers, and duplicates. Data cleaning is a crucial step in data preprocessing to alleviate the potential negative impact of such issues on the quality of the dataset. In this study, we dropped all samples that contained Nan, empty, or infinite values. In this study, after data cleaning, the sample count was reduced from 215,761 to 215,076, with 685 removed samples.

#### 4.1.3. Data Transformation

Due to the varying scales present in the dataset, this study utilized MinMax scaling to bring all features to a standard scale. MinMax scaling is a linear transformation applied to the original data. Given the original data as x and the transformed data as x′, the scaling formula is as follows:

(12) $$x\prime =\frac{\left(x-min\right)}{\left(max-min\right)}$$

Here, min and max refer to the minimum and maximum values of the column in which x resides.

This standardization method has broad applicability. By utilizing this method, data are mapped to a range of $\left[0,1\right]$ while maintaining the original structure of the data, which is unlike the Z-Score standardization method. As a result, this approach enables faster and simpler data normalization while falling within a specific range.

#### 4.1.4. Feature Extraction

In this study, an improved binary grey wolf optimization algorithm was utilized for feature selection to select the optimal subset of features from multiple feature subsets. The following section will provide details on the improvements made.

**1.**  **Initialization based on chaotic mapping algorithm**

In population-based optimization algorithms, pseudo-random number generators are typically used to initialize the population, which can lead to uneven distribution and a reduction in diversity and roaming speed. To address this issue, this study utilized a chaotic mapping algorithm to optimize the initial positions of the GWO algorithm, promoting faster convergence. This approach maintains population diversity and provides an even distribution of the initial population. Specifically, the chaotic parameters were first linearly mapped to the exploration space. Afterwards, the chaotic transformation was employed to achieve the exploratory objective. This method possesses both chaotic randomness and traversing abilities, thereby avoiding the entrapment of local optima initialization based on a chaotic mapping algorithm.

Logistic and Tent mapping are commonly used chaotic techniques [32]. Tent mapping provides better uniformity and convergence speed than Logistic mapping. Therefore, in this study, we utilized Tent mapping. The specific formula is as follows:

(13) $$x_{n+1}=f\left(x_{n}\right)=\left\{\begin{array}{c} \frac{x_{n}}{\alpha },x_{n}\in \left[0,\left.\alpha \right)\right.\\ \frac{\left(1-x_{n}\right)}{\left(1-\alpha \right)},x_{n}\in \left[\alpha ,1\right] \end{array}\right.$$

**2.**  **Setting of Nonlinear Search Parameters**

In the traditional GWO algorithm, the convergence factors linearly decrease with the number of iterations, somewhat limiting the population’s exploratory ability after half of the iterations. However, excessive searching increases randomness and may result in suboptimal results, while insufficient searching may cause entrapment in local optima. Therefore, selecting an appropriate search duration is crucial. To address this issue, a nonlinear adjustment method was proposed in [33] to better balance the exploratory and exploitative abilities of the GWO algorithm. Taking inspiration from this approach, in this study, we improved upon it by utilizing a cosine function to adjust the convergence factor, enhancing the algorithm’s global search ability. The specific formula for this computation is as follows:

(14) $$a_{Alpha}=2\cos\left(\frac{\pi }{2}\times {\left(\frac{t}{T}\right)}^{2}\right)$$

(15) $$a_{Beta}=2\cos\left(\frac{\pi }{2}\times \left(\frac{t}{T}\right)\right)$$

(16) $$a_{Delta}=1+e^{\left(\frac{t}{T}-1\right)}$$

**Figure 5:**
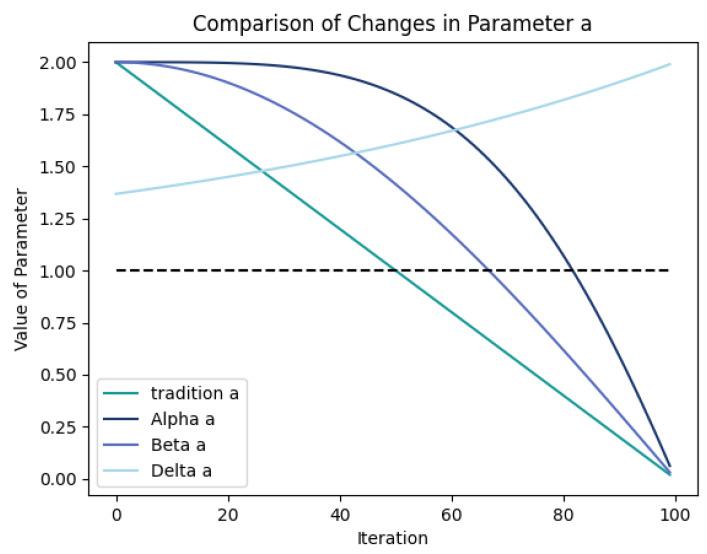
Comparison of improved and standard convergence factors.

In order to better optimize the search performance of the Alpha, Beta, and Delta wolves in the GWO algorithm, different search parameters a were designed, as shown in Figure 5. Specifically, $a_{Alpha}$ extends the time when $\left|A\right|$ is greater than 1 to keep it more significant than 1 during the middle and later stages of the iterations.

This means that utilizing this cosine nonlinear convergence factor can help the population maintain longer exploratory abilities and have more opportunities to escape from local optima. As the second-best solution, Beta wolf was used to guide the population to explore the search space around it. Therefore, $a_{Beta}$ was designed to make $\;\left|A\right|$ enter $\left[0,1\right]\;$ earlier than $\left|A\right|$ of the Alpha wolf. When the population tends to move towards the Beta wolf during updating, the Beta wolf can guide the population to explore the search space around it. As the third-best solution, $a_{Delta}$ was designed to maintain $\left|A\right|$ greater than 1, guiding the population to maintain a certain distance and better search for prey throughout the entire area.

**3.**  **Improve position updating method**

In traditional GWO, the position updating method is relatively simplistic, which cannot guarantee that Alpha, the optimal solution, maintains its guiding role throughout the process. To address this issue, we introduced a modified position updating Equation (17), wherein we incorporated weight factors $\omega_{1}>\omega_{2}>\omega_{3}$, with respective values of 6, 3, and 2 (these values can be adjusted as needed to obtain optimal results). This ensured that Alpha remained in the guiding position throughout the updating process.

(17) $$\vec{X}\left(t+1\right)=\frac{\left(\omega_{1}\cdot \vec{X_{1}}+\omega_{2}\cdot \vec{X_{2}}+\omega_{3}\cdot \vec{X_{3}}\right)}{\omega_{1}+\omega_{2}+\omega_{3}},\omega_{1}>\omega_{2}>\omega_{3}$$

**Figure 6:**
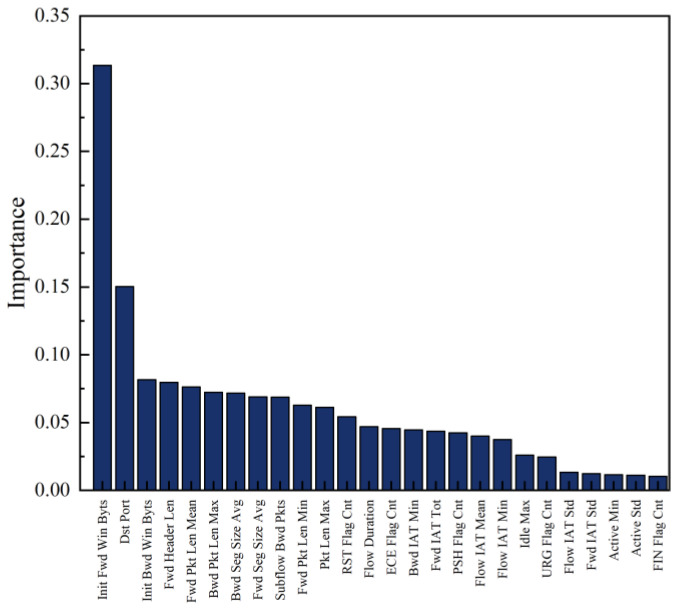
The importance of each feature.

In total, 26 features were selected from the original dataset of 79 features using the algorithm mentioned above. The pseudo-code of our algorithm is shown in Algorithm 1. The results are displayed in Table 2. Furthermore, the algorithm’s selection process also identified the importance of each selected feature, which is illustrated in Figure 6.
**Algorithm 1:** Improved binary grey wolf optimization algorithm.**Input:** n Number of grey wolves in the pack,   Iter Number of iterations for optimization. **Output:**
$x_{\alpha }$ Optimal grey wolf binary position,     $\;f\left(x_{\alpha }\;\right)$ Best fitness value. 1: **Begin** 2: Initialize the population according to Equation (13). 3: Calculate the fitness value of the group and find $x_{\alpha },x_{\beta }$ and $x_{\delta }$. 4: **While** (t < Iter): 5:  **For** each ${wolf}_{i}$ population: 6:  Update ${wolf}_{i}$ position to a binary position according to Equations (9) and (17) 7:  **end for** 8:  Update a according to Equations (14)–(16). 9:  Update A and C according to Equations (3) and (4). 10: Evaluate the positions of individual wolves according to Equation (11). 11: Update $x_{\alpha }$, $x_{\beta }$ and $x_{\delta }$ according to Equations (7) and (8) 12: t = t + 1 13: **end while** 14: **return**
$x_{\alpha }$ 15: **End**

### 4.2. Classifiers Used

In machine-learning-based intrusion detection systems, the primary objective is to determine the normality or abnormality of network traffic, necessitating the training of machine learning algorithms. Several machine learning algorithms are available in the literature [34]. Therefore, SVM, RF, Decision Tree, XGBoost, and k-NN were used in this study. In addition, performance evaluation of these classifiers was required to deploy the best classifier within the controller for DDoS attack detection. An overview of each classifier is as follows:

#### 4.2.1. Support Vector Machines (SVMs)

**Figure 7:**
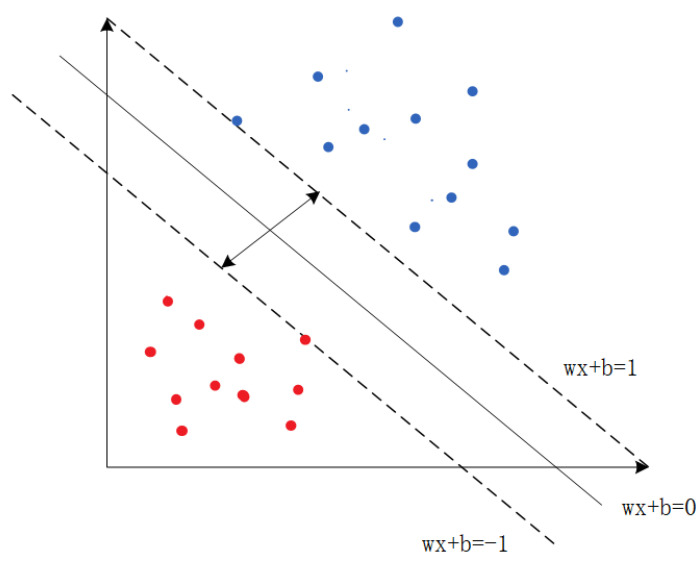
Support Vector Machine (SVM).

SVM is a binary classification model that separates data points of different categories by searching for a hyperplane (a straight line in 2D or a flat plane in 3D). Taking linearly separable data as an example, as shown in Figure 7, SVM aims to find the closest points to the hyperplane, which are called support vectors. In the figure, the red points represent negative instances, and the blue points represent positive instances. Support vectors are the points that satisfy the constraints.

(18) $$y_{i}\left(\omega \cdot x_{i}+b\right)-1=0$$

If the support vectors for positive instances of $y_{i}=1$ lie on the following hyperplane:

(19) $$H_{1}:\;\omega \cdot x+b=1$$

For the negative instances of $y_{i}=-1$, the support vectors lie on the following hyperplane:

(20) $$H_{2}:\;\omega \cdot x+b=-1$$

The Support Vector Machine consists of $H_{1}$ and the data points on $H_{2}$. The margin boundary between $H_{1}$ and $H_{2}$ is 2/‖ω‖. To achieve the maximum margin classification hyperplane, the problem that needs to be solved can be formulated as a constrained optimization problem, as shown below:

(21) $$\underset{w,b}{\min}\frac{1}{2}{\left|\left|\omega \right|\right|}^{2}$$

(22) $$y_{i}\left(\omega \cdot x_{i}+b\right)-1\geq 0,i=1,2,3,\cdots ,N$$

SVM has exhibited remarkable performance in solving classification problems, and many researchers have applied it to attack detection and classification. Furthermore, SVM has several commonly used kernel functions that map low-dimensional data into high-dimensional feature spaces, resulting in linearly separable data. This feature has been used in many works for attack detection [35,36,37,38].

#### 4.2.2. Random Forest (RF)

**Figure 8:**
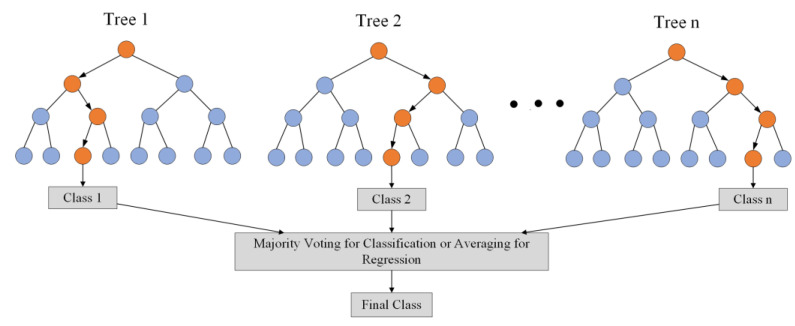
Random Forest (RF).

RF is an ensemble model comprising many decision trees, each trained on a different subset of the dataset. Although each decision tree is a weak classifier, they can achieve high accuracy when used together. When classifying or predicting new data samples using RF, each decision tree classifies the data based on its features, and the final result is obtained by either averaging or taking the majority vote, as shown in Figure 8. During the training process, each decision tree in RF undergoes independent sampling using Bootstrap and randomized feature selection. This approach avoids overfitting and improves the model’s stability and generalization capacity. Due to these characteristics, an increasing number of researchers use RF for both attack detection and regression problems [6,39,40].

#### 4.2.3. Decision Trees

The Decision Tree algorithm is a widely used machine learning technique that enables traversal from a root to its leaves. It functions on a fundamental principle of creating a tree-structured model by splitting the dataset, where each node represents a feature, each diverging branch conveys a feature’s distinct value, and the final leaf node represents a definitive classification result. The advantages of decision trees include their simplicity in understanding and interpretation, ability to handle nonlinear features, suitability for large datasets, and extension to multi-class problems. Furthermore, decision trees can be used as an ensemble model with other machine learning techniques to improve classification accuracy. Thus, decision trees are typically optimal for classification models in diverse application scenarios. Within the DDoS detection field, decision trees have also secured widespread preference [16,41,42,43].

#### 4.2.4. XGBoost

The XGBoost algorithm is an ensemble learning technique based on the concept of the Gradient Boosting Tree. It trains a group of weak classifiers, such as decision trees, iteratively enhancing the model’s predictive ability and ultimately constructing a robust classifier. This method builds each tree on the residual error of the previous tree while minimizing the loss function (e.g., mean squared error). XGBoost improves model accuracy and efficiency by optimizing the Gradient Boosting Tree algorithm, staffed with regularization methods and parallel processing to avoid overfitting and accelerate training speed. The algorithm’s success stems from its exceptional accuracy and speed, rendering it suitable for attack detection in several research works [44,45]. This study evaluated its effectiveness against other classifiers concerning DDoS attack detection in SDNs.

#### 4.2.5. K-Nearest Neighbors (k-NN)

The k-NN algorithm is grounded on instance-based learning, enabling it to classify and regress without definite assumptions about the underlying data distribution. The k-NN algorithm assigns a data point to one of its k-closest neighbors, selecting the majority class vote amongst its k-nearest neighbors during classification. Subsequently, the algorithm determines the category to which a new point belongs based on its nearest neighbors in classification problems. The classification of unknown instances is determined via the following steps:

1. Calculate the distance between the sample to be classified and each sample in the training set (usually using the Euclidean distance or Manhattan distance).
2. Select the K available class samples closest to the sample to be classified in ascending order of distance.
3. Determine the category of the sample to be classified based on the categories of these K samples through majority voting. It is usually preferred to select an odd number for K to avoid tied votes.

(23) $$d_{euclidean}=\sqrt{\sum_{i=1}^{n}{\left(x_{i}-y_{i}\right)}^{2}}$$

(24) $$d_{manhattan}=\sum_{i=1}^{n}\left|x_{i}-y_{i}\right|$$

The k-NN algorithm presents a range of advantages, including simplicity, ease of understanding, and independence from pre-trained models. It addresses classification and regression challenges effectively and delivers remarkable performance with nonlinearly separable data. Its practicality and simplicity have led to widespread use, particularly in resolving multi-class classification issues. Furthermore, the algorithm has been acknowledged via numerous research works for its outstanding performance in attack detection scenarios [25,46,47].

### 4.3. Mitigation Strategies

The primary function of the Mitigation module is to prevent harmful packets within the network, which can be achieved through the use of the pre-existing SDN functionalities. The detected host that belongs to an attacker must be identified, after which traffic modification rules become installed within the switch’s flow table to discard packets emanating from the attachment point ($a_{dpid}$, $\alpha_{\mathrm{inport}}$). Algorithm 2 outlines the pseudocode utilized to execute the Mitigation procedure.
**Algorithm 2**: Mitigation procedure.1: **Begin** 2: get corresponding Datapath 3: set matching criteria with respect to $\alpha_{\mathrm{inport}}$ 4: send a flow modification message to switch 5: drop packets corresponding to $\alpha_{\mathrm{inport}}$ of $a_{dpid}$ 6: **End**


## 5. Experiments and Analysis of Results

This section presents details of how the experiments were conducted, the presentation of the results, and the comparison and discussion with other similar works.

### 5.1. Experimental Environment

The study employed Ubuntu 18.04 operating system with 16 GB RAM, AMD Ryzen 7 5800 H processor, clocked at 3.20 GHz. Mininet was employed in the working environment and linked to the Ryu remote controller. Ryu was selected as the SDN controller due to its superior performance to other related controllers [48]. Integration of Ryu with other Python libraries used in the study was straightforward due to its development in Python. The experiment and performance evaluation process used libraries such as NumPy, Matplotlib, Scikit-learn, and Panda.

### 5.2. Evaluation Criteria

**Figure 9:**
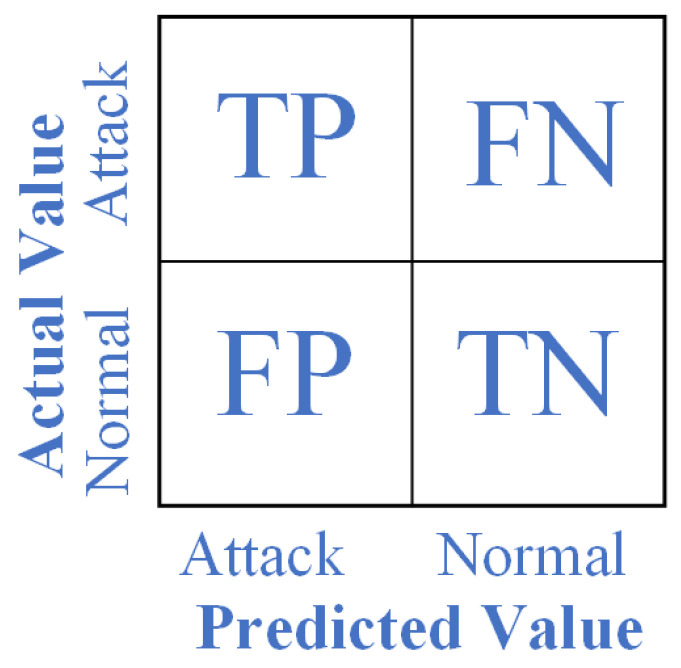
Confusion matrix.

The prediction results can be divided into four categories: True Positive (TP), False Positive (FP), True Negative (TN), and False Negative (FN), as shown in Figure 9. TP represents the number of attack samples correctly predicted as attack samples by the algorithm, TN represents the number of normal samples correctly predicted as normal samples, while FP represents the number of normal samples incorrectly predicted as attack samples by the algorithm. FN represents the number of attack samples incorrectly predicted as normal samples by the algorithm. In this study, the following indicators were used to evaluate the proposed model:

1. **Accuracy:** Accuracy refers to the proportion of correctly predicted samples compared with the total number of samples in the prediction process.
  (25) $$A\mathrm{ccuracy}=\frac{TP+TN}{TP+TN+FP+FN}$$
2. **Precision:** Precision is defined as the percentage of samples correctly predicted as positive out of all samples predicted as positive.
  (26) $$Precision=\frac{TP}{TP+FP}$$
3. **Recall:** Recall is the proportion of true positive samples to all positive samples.
  (27) $$Recall=\frac{TP}{TP+FN}$$
4. **F1 score:** F1 score is a metric used to evaluate the accuracy of optimistic class predictions. It represents the ratio of correctly identified positive samples to all samples predicted as positive.
  (28) $$\mathrm{F1-Score}=\frac{2\cdot Recall\cdot Precision}{Recall+Precision}$$
5. **ROC curve:** The ROC curve is a graphical representation of a model’s classification performance, with better-performing models having higher curves and larger areas underneath. Typically, the AUCROC is used to evaluate model performance based on the area under the ROC curve. A value of one indicates near-perfect classification, while lower values indicate poorer model performance.

### 5.3. Analysis of Results

#### 5.3.1. Performance of Each Classification Model

**Figure 10:**
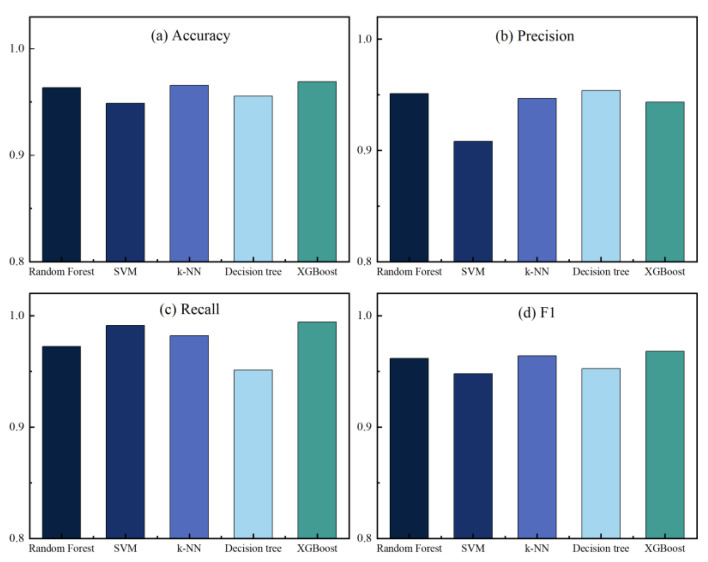
Performance indicators of each classifier in the original dataset.

**Figure 11:**
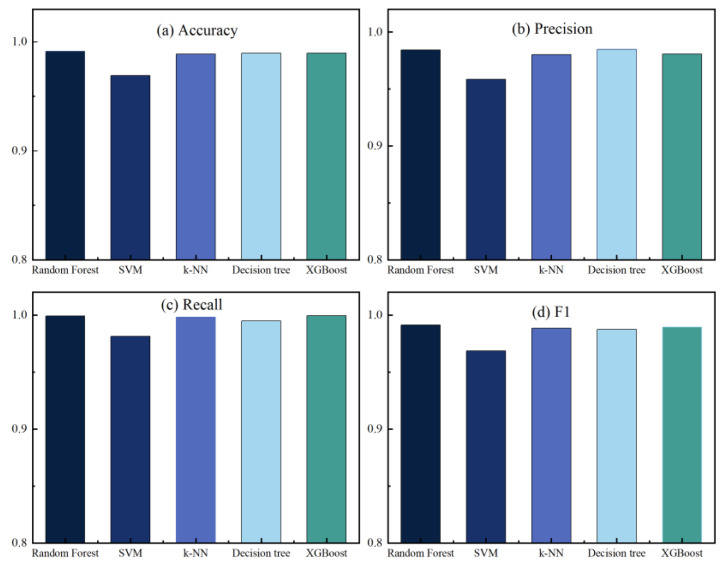
Performance indicators of each classifier in the dataset after feature extraction.

In this work, we have implemented five machine learning algorithms: RF, SVM, k-NN, Decision Tree, and XGBoost. All classification algorithms used default parameters and were trained and tested using K-fold cross-validation. According to work performed in the literature [49,50], a value of 10 for K is preferred. Figure 10 and Figure 11 show the performance metrics of each classifier on the original dataset and the dataset after performing feature extraction, respectively, where (a) accuracy, (b) precision, (c) recall, and (d) F1 score. See Table 3 for details of the classification results.

**Figure 12:**
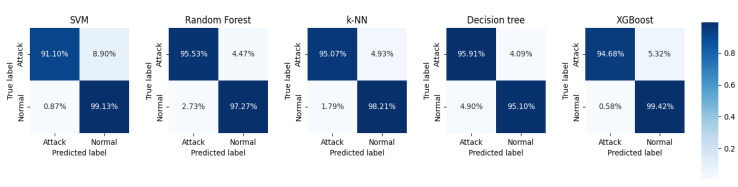
The confusion matrix of each classifier on the original dataset.

**Figure 13:**
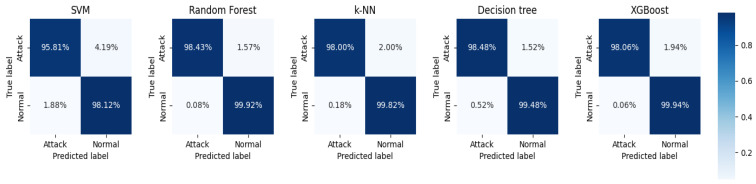
The confusion matrix of each classifier on the dataset after feature extraction.

**Figure 14:**
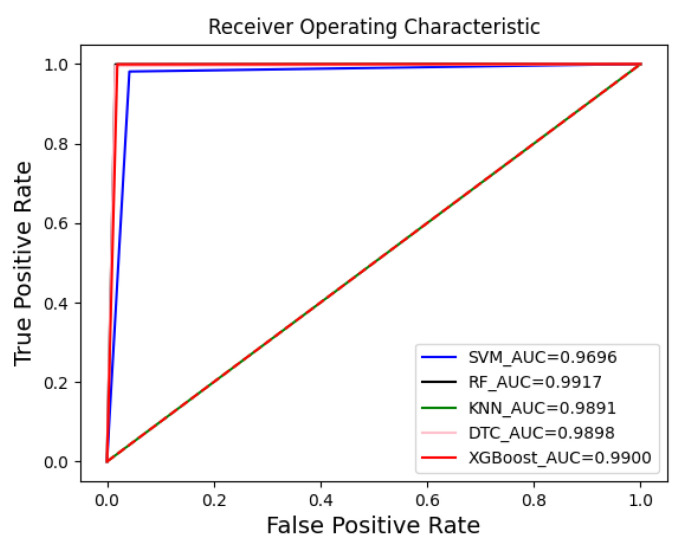
AUC-ROC curves for all classifiers.

From the data analysis, we can see that the XGBoost algorithm had the best performance on the original dataset with an accuracy of 0.969. However, it was slightly less accurate than RF but had the best overall performance. After feature extraction, the accuracy of each classifier improved to a greater or lesser extent, with the Decision Tree algorithm improving the most, by 0.0341, followed by the RF algorithm by 0.0278, and the SVM algorithm, by the least, by 0.0202. Overall, except for the accuracy, which was slightly lower than that of the Decision Tree algorithm, the RF algorithm performed the best. The RF algorithm performed the best, except for the accuracy, which was slightly lower than that for the Decision Tree algorithm, and all other metrics were better than the other algorithms. In addition, Figure 12 and Figure 13 show the confusion matrix for each classifier on the original dataset and the dataset after performing feature extraction, respectively. The misclassification rate of each classifier decreased, with the RF classification algorithm performing the best. The RF algorithm performed the best in misclassifying normal traffic as attack traffic and misclassifying attack traffic as normal traffic. The AUC-ROC curves for all classifiers are presented in Figure 14, where SVM_AUC is 0.9696, RF_AUC is 0.9917, k-NN_AUC is 0.9891, DTC_AUC is 0.9898, and XGBoost_AUC is 0.99. Therefore, we consider the RF algorithm to be the best performer.

#### 5.3.2. Performance in DDoS Attack Detection

**Figure 15:**
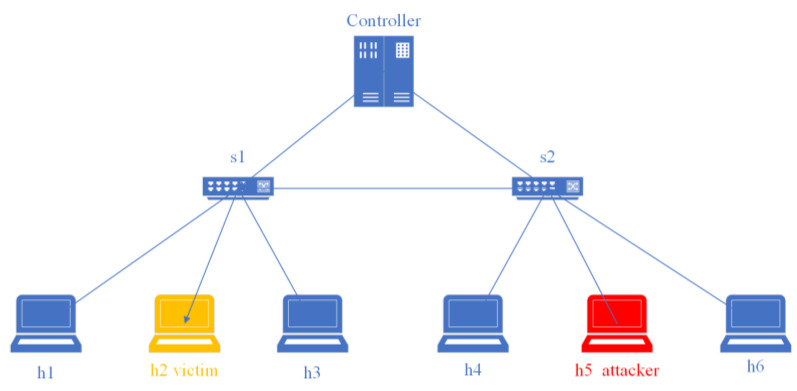
Network topology.

**Figure 16:**
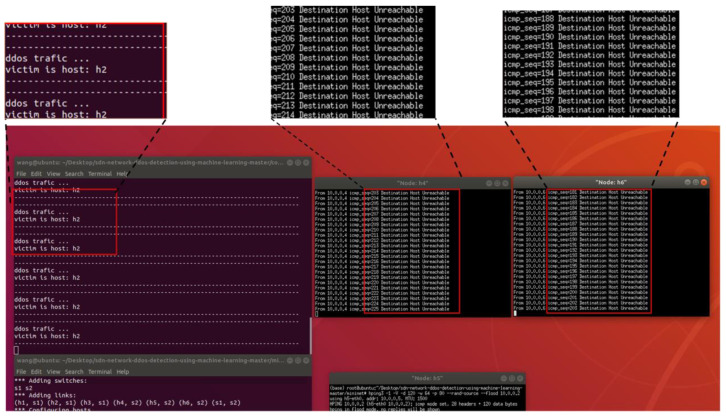
Response of the controller to DDoS attacks.

**Figure 17:**
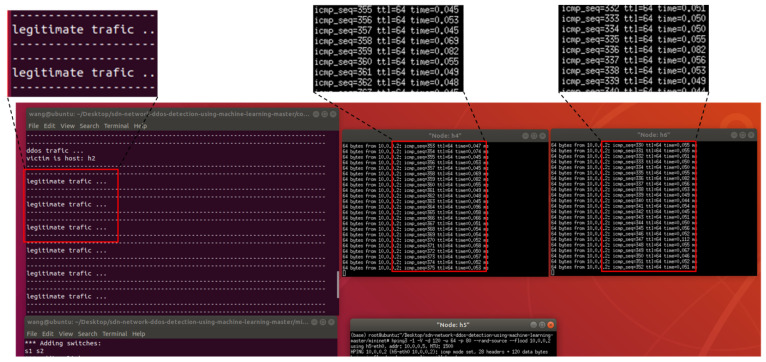
Mitigating controller response after DDoS attacks.

Figure 15 shows the network topology for this experiment, which includes a controller, two switches, and six hosts. h5 is the attack initiator, h2 is the victim, and h4 and h6 send normal traffic requests to h2. We first preprocessed the network traffic to extract the required features and then used the best-performing classification algorithm for DDoS attack detection. A notification was sent to the user if the traffic was identified as an attack; otherwise, the flow table was issued normally. As shown in Figure 16, when we used hping3 to launch a flooding attack on h2 using host h5, h4 and h6 could not send data requests to h2, and the controller alerted the user that h2 had received the attack. When the mitigation strategies (Algorithm 2) were activated, the traffic rule was configured to drop packets corresponding to a specific input port to achieve mitigation of the flood attack, and h4 and h6 could send data to h2 normally, as shown in Figure 17.

#### 5.3.3. Comparison with Other Work

Table 4 compares the work in this paper with other similar works.

Polat et al. [14] used Filter, Wrapper, and Embedded three wrapper feature selection algorithms paired with SVM, NB, ANN, and k-NN classification models for training and testing, respectively. The results show that the Wrapper-based feature extraction and k-NN classifier performed best with accuracy, precision, recall, and f1 scores of 0.983, 0.9772, 0.9773, and 0.977, respectively. Compared with them, the work in this paper improved in accuracy, precision, recall, and f1 score by 0.0083, 0.0071, 0.0219, and 0.0143.

Aamir et al. [17] used before-and-after elimination, Chi2, and information gain score methods for feature selection. Then, they used different supervised machine learning models for classification, where the combination of Chi2 and k-NN performed the best with an accuracy of 0.9351. However, their data preprocessing process was tedious, and other evaluation criteria were not given. The accuracy of this work was improved by 0.0562 compared with their work.

Polat et al. [21] used LSTM and GRU in parallel for feature extraction. Then, SVM was used for classification detection with an accuracy of 0.9762, a precision of 0.9772, recall of 0.9679, and F1 score of 0.9719. Unfortunately, the feature extraction process and the extraction results were not described. Compared with this work, the accuracy, precision, recall, and F1 score were improved by 0.0083, 0.0071, 0.0219, and 0.0143, respectively.

Thangasamy et al. [22] used a deep trust network for feature extraction and then used PSO-LSTM for classification detection with an accuracy of 0.98, a precision of 0.97, recall of 0.95, and F1 score of 0.96. Although they used a feature extraction algorithm, they did not mention the specific features extracted. The work in this paper remedies this deficiency with an improvement of 0.0113, 0.0143, 0.0492, and 0.0313 in accuracy, precision, recall, and F1 score, respectively, compared with theirs.

Ahuja et al. [26] generated UDP, TCP, and ICMP attacks and regular traffic, then extracted 23 features. Out of 23 features, they selected 8 features. The classification’s accuracy, recall, precision, and F1 score using the hybrid machine learning model of SVM-RF were 0.988, 0.9827, 0.979, and 0.9765, respectively. The improvement of this paper’s work in accuracy, precision, recall, and F1 score are 0.0033, 0.0016, 0.0202, and 0.0148, respectively.

The improved binary grey wolf optimized feature extraction method proposed in this paper performs better with the Random Forest model. It should be noted that similar studies in the literature use different datasets and models. Therefore, justification of the comparison results is difficult. The literature [14,17,21,22,26] did not show the performance of their model for DDoS attack detection on SDNs and did not develop mitigation strategies. In contrast, the model in this paper can accurately detect DDoS attacks on SDNs to give users hints and successfully mitigate the attacks.

## 6. Conclusions and Future Work

This study proposes a DDoS detection method based on feature engineering and machine learning in SDN. The method is divided into the feature extraction and model selection module and the DDoS attack detection module. In module 1, we used an improved binary grey wolf optimization algorithm for feature extraction. We used five machine learning models—RF, SVM, XGBoost, Decision Tree, and k-NN—to evaluate and select the best classifier for the original and feature-extracted datasets, respectively. The results showed that the highest accuracy of XGBoost was 0.969 on the original dataset; after feature extraction, the number of features on the dataset changed from 79 to 26, and although there were fewer features, all the classifiers improved in all metrics. Among them, the RF classifier performed best under accuracy, precision, recall, and f1_score metrics with 0.9913, 0.9843, 0.9992, and 0.9913, respectively. In Module 2, we deployed the best classifier selected in Module 1 to the controller and performed DDoS detection using features from a subset of the best features. The results show that the proposed method can detect DDoS attacks and alert users.

In our future work, we intend to enhance and expand the current methods for detecting DDoS attacks. This includes utilizing advanced feature-engineering techniques and machine learning models, such as Deep Learning, to improve the classifier’s performance and accuracy. We will also research how to mitigate adversarial attacks to increase the method’s robustness and adaptability. Additionally, we plan to integrate this method with other network security technologies to create a more comprehensive and complete network security solution.

## Figures and Tables

**Table 1:** Literature survey.

References	Dataset	Feature Selection Methods	Class Method
[14]	Synthetic	Filter-Based, Wrapper-Based, Embedded-Based	SVM, NB, ANN, k-NN
[15]	NSL-KDD	Genetic Algorithm	RBF neural network optimized by cuckoo search
[16]	CIC-DDoS2019	Extra Tree Classifier	RF, SVM, NB, Decision Tree, XGBoost, AdaBoost
[17]	DS00_Full	*p*-value (t-statistic test), Chi-square test, and Information gain test	k-NN, NB, SVM, RF, ANN
[18]	CIC-DDoS2019	A novel hybrid metaheuristic optimization algorithm (BHO)	Ensemble employs six base classifiers (two SVMs, two RF, and two Gradient Boosted Machines)
[19]	CIC-DDoS2019	The info gain attribute evaluation algorithm	Based on Deep Neural Networks (DNN), Convolutional Neural Networks (CNN), and Long Short-Term Memory (LSTM)
[20]	CSE-CIC-IDS2018	Synthetic Minority Oversampling Technique	k-NN, RF, Gradient Boosting, AdaBoost, Decision Tree, and Linear Discriminant Analysis algorithms
[21]	Synthetic	LSTM and GRU	SVM
[22]	NSL-KDD	Deep belief network feature extraction	PSO-LSTM model
[10]	Synthetic	Grey wolf optimization algorithm and CNN	Optimized LSTM
[23]	Synthetic	5 features are extracted from the dataset	Decision Tree, Deep Learning (DL), k-NN, Logistic Regression (LR), RF, and SVM
[24]	Synthetic	7 features are extracted from the dataset	SVM, RF, k-NN, XGBoost, NB
[25]	Synthetic	4 features are extracted from the dataset	One method adopts the degree of DDoS attack and improved k-NN
[26]	Synthetic	23 features extracted, 8 selected from the extracted features	SVC

**Table 2:** An overview of the extracted features.

Feature	Explanation
Dst Port	Destination Port
Init Fwd Win Byts	Number of bytes sent in initial window forward direction
Init Bwd Win Byts	Number of bytes sent in initial window backward direction
Fwd Header Len	Total length for forward headers in bytes
Pkt Len Max	Maximum length of a packet
Bwd Pkt Len Max	Maximum size of backward packets
Fwd Pkt Len Mean	Mean size of packet in forward direction
Bwd Seg Size Avg	Average size observed backward direction
Fwd Seg Size Avg	Average size observed forward direction
Subflow Bwd Pkts	The average number of packets in a sub flow in the backward direction
Fwd Pkt Len Min	Minimum size of packet in forward direction
Flow Duration	Duration of the flow (microsecs)
ECE Flag Cnt	Number of packet switch ECE flags
Bwd IAT Min	Minimum time between two packets sent in the backward direction
RST Flag Cnt	Number of packet switch RST flags
Flow IAT Mean	Mean time between two packets sent in the flow
Fwd IAT Tot	Total time between two packets sent in the forward direction
PSH Flag Cnt	Number of packet switch PSH flags
Flow IAT Min	Minimum time between two packets sent in the flow
Idle Max	Maximum time a flow was idle before becoming active
URG Flag Cnt	Number of packet switch URG flags
Flow IAT Std	Standard deviation time between two packets sent in the forward direction
Fwd IAT Std	Standard deviation time between two packets sent in the forward direction
Active Min	Minimum time a flow was active before becoming idle
Active Std	Standard deviation time a flow was active before becoming idle
FIN Flag Cnt	Number of packet switch FIN flags

**Table 3:** Comparing performance indicators among various classifiers for the original dataset and the dataset post feature extraction.

	Dataset before Feature Extraction				Dataset after Feature Extraction			
	Accuracy	Precision	Recall	F1	Accuracy	Precision	Recall	F1
RF	0.9635	0.951	0.9723	0.9616	0.9913	0.9843	0.9992	0.9913
SVM	0.9487	0.9082	0.9913	0.9479	0.9689	0.9583	0.9812	0.9685
XGBoost	0.969	0.9432	0.9942	0.968	0.9894	0.9806	0.9994	0.9894
k-NN	0.9655	0.9466	0.9821	0.964	0.9886	0.9801	0.9982	0.9885
Decision Tree	0.9554	0.9537	0.9511	0.9525	0.9895	0.9847	0.9947	0.9875

**Table 4:** Comparison with other studies.

References	Year	Model	Accuracy	Precision	Recall	F1_Score
[14]	2020	Wrapper-Based and k-NN	0.983	0.9772	0.9773	0.9770
[17]	2019	Chi2 and k-NN	0.9351	NA	NA	NA
[21]	2022	Parallel RNN-based SVM Model	0.9762	0.9772	0.9679	0.9719
[22]	2023	Deep belief network feature extraction and PSO-LSTM	0.98	0.97	0.95	0.96
[26]	2021	SVC-RF	0.988	0.9827	0.979	0.9765
Our study	2023	Improved binary grey wolf optimization algorithm and RF	0.9913	0.9843	0.9992	0.9913

## Data Availability

Not applicable.
